# Isolation and Characterization of Human Intestinal Bacteria *Cytobacillus oceanisediminis* NB2 for Probiotic Potential

**DOI:** 10.3389/fmicb.2022.932795

**Published:** 2022-07-13

**Authors:** Monika Yadav, Tarun Kumar, Akshay Kanakan, Ranjeet Maurya, Rajesh Pandey, Nar Singh Chauhan

**Affiliations:** ^1^Department of Biochemistry, Maharshi Dayanand University, Rohtak, India; ^2^Integrative GENomics of Host-PathogEn (INGEN-HOPE) Laboratory, Council of Scientific and Industrial Research-Institute of Genomics and Integrative Biology (CSIR-IGIB), New Delhi, India; ^3^Academy of Scientific and Innovative Research (AcSIR), Ghaziabad, India

**Keywords:** probiotic, human gut microbe, microbiome therapeutics, microbial characterization, microbial isolation

## Abstract

Systemic characterization of the human gut microbiota highlighted its vast therapeutic potential. Despite having enormous potential, the non-availability of their culture representatives created a bottleneck to understand the concept of microbiome-based therapeutics. The present study is aimed to isolate and evaluate the probiotic potential of a human gut isolate. Physiochemical, morphological, and phylogenetic characterization of a human gut isolate identifies it as a rod-shaped gram-negative microbe taxonomically affiliated with the *Cytobacillus* genus, having an optimal growth at 37°C in a partially alkaline environment (pH 8.0). This human gut isolate showed continuous growth in the presence of salts (up to 7% NaCl and 10% KCl), antibiotics, metals and metalloids [silver nitrate (up to 2 mM); lead acetate (up to 2 mM); sodium arsenate (up to 10 mM); potassium dichromate (up to 2 mM)], gastric and intestinal conditions, diverse temperature (25–50°C), and pH (5–9) conditions making it fit to survive in the highly variable gut environment. Genomic characterization identified the presence of gene clusters for diverse bio-catalytic activity, stress response, and antimicrobial activity, as well as it indicated the absence of pathogenic gene islands. A combination of functional features like anti-amylase, anti-lipase, glutenase, prolyl endopeptidase, lactase, bile salt hydrolase, cholesterol oxidase, and anti-pathogenic activity is indicative of its probiotic potential in various disorders. This was further substantiated by the CaCo-2 cell line assay confirming its cellular adherence and biosafety. Conclusively, human gut isolate possessed significant probiotic potential that can be used to promote animal and human health.

## Introduction

Human gut microbes play an important role in the maintenance of human health through active participation in host metabolism, immunity, gut homeostasis, and pathogen eradication (Yadav et al., 2018; Yadav and Chauhan, 2022). Gut microbes are being characterized for their therapeutic potential to treat human disorders (Thaiss and Elinav, 2017). *Christensenella* sp. is shown to reduce depression and anxiety-like behavior (Verma et al., 2020). *Akkermansia muciniphila* augments relief to the host from metabolic disorders (Kalia et al., 2022a), as well as protects against atherosclerosis by reducing gut permeability and preventing inflammation (Li et al., 2016). *Lactobacillus johnsonii* protects the host against the onset of cancer (Marcial et al., 2017). *Bifidobacterium longum* reduces the severity of Crohn's disease (Yao et al., 2021) and repairs the mucus layer integrity impaired due to a high-fat diet (Schroeder et al., 2018). *Oxalibacterium formigenes* prevent kidney stones by ensuring oxalic acid breakdown (Jalanka-Tuovinen et al., 2011). *Bacteroides* sp. protects against adiposity (Walker and Parkhill, 2013). *Lactobacillus johnsonii, Akkermansia muciniphila, Bifidobacterium longum, Bacteroides* sp., *Roseburia intestinalis, Faecalibacterium prausnitzii*, and *Bacillus* sp. are characterized for certain health-promoting benefits such as anti-cancer, anti-diabetic, anti-obesity, anti-pathogenic, as well as cholesterol-removing properties (Aswathy et al., 2008). These studies highlighted the scope of harnessing the potential of gut microbes as probiotic strains in disease therapeutics (Yadav and Chauhan, 2022). The impact of probiotics on human as well as animal health has promoted their use as food additives even on a commercial scale (Cuello-Garcia et al., 2015; Varankovich et al., 2015). These strains are also being used as food additives to improve the health of poultry animals for disease prevention and increased meat production (Kalia et al., 2022b). Several strains of *Lactobacillus, Bifidobacterium*, and *Bacillus* have also been used as potential probiotics (Lee et al., 2019). *B. cereus, B. clausii, B. coagulans, B. licheniformis, B. polyfermenticus, B. pumilus*, and *B. subtilis* are well-characterized commercial probiotic strains (Lee et al., 2019). Despite the enormous therapeutic potential, the majority of human gut microbes could not be exploited for their probiotic potential, which is attributed to the lack of their cultured representative. Scientific explorations are required to culture human gut microbes to harness their probiotic potential. The current study was designed to culture a human gut bacterium and characterize it for its probiotic potential. The probiotic potential of this microbe can be efficiently used to improve animal as well as human health.

## Methods

### Sample Collection and Ethical Statement

Bacterial isolate strain NB2 was cultured from a fecal sample collected from a healthy individual (age 28 years, female, BP 120/80, blood sugar 100–120 mg/dl, BMI 26.4, with no symptoms of any illness). A total of 100 mg of fecal sample was homogenized and serially diluted (10^−1^-10^−5^) in phosphate buffer saline (pH 7.4). About 100 μl of each serial dilution was plated on a nutrient agar medium plate. The culture plates were incubated at 37°C till the appearance of microbial colonies. Sub-culturing of microbial colonies was performed in LB (Luria Bertini) agar medium at 37°C. The study was conducted after receiving ethical clearance from the Human ethical committee at M. D. University, Rohtak Haryana, India. Strict human ethical guidelines were followed, and written consent was sought from the individual included in this study.

### Molecular and Phenotypic Characterization

Gram staining of bacterial isolate strain NB2 was performed using a commercially available gram-staining kit (Himedia, K001-1KT). The morphology was observed using a compound microscope. The microbial growth pattern was analyzed after continuously culturing the bacterial isolate strain NB2 (0.01 at OD_600nm_) in LB broth for 24 h at 37°C. Taxonomic affiliation of human gut isolate NB2 was performed using 16S rRNA gene analysis (Kumar Mondal et al., 2017). The substrate preference of bacterial isolate strain NB2 was checked using the Hi-Carbo kit (Himedia, KB009A-1KT, KB009B1-1KT, KB009C-1KT) at 37°C for 24 h following the manufacturer's instructions.

### Genome Characterization of Bacterial Isolate Strain NB2

The genomic DNA of bacterial isolate strain NB2 was sequenced on Illumina MiSeq using Nextera XT DNA Library Prep kit following the manufacturers' protocol (https://sapac.illumina.com/content/dam/illumina-marketing/documents/products/datasheets/datasheet_nextera_xt_dna_sample_prep.pdf). Raw reads were quality checked using FASTQC v0.11.9 (http://www.bioinformatics.babraham.ac.uk/projects/fastqc) and fastQ Validator v0.1.1 (https://github.com/statgen/fastQValidator). Removal of contaminated reads was performed to get the error corrected reads. The SPAdes v3.15.1 assembler was used for the *de novo* assembly which uses an automatic k-mer optimization approach and is thereby a good tool for bacterial genome assembly. It uses Bayes Hammer to perform read error correction on each data set and Mismatch Corrector, a post-processing tool, to reduce the number of mismatches in assembly using the BWA-0.7.17 tool. Further, the BUSCO v5.0.0 assessment tool was used with the latest bacterial orthologous catalog (bacteria_odb10) for analyzing the completeness of a set of predicted genes in bacterial genome assemblies (https://busco.ezlab.org/). The sequenced genome was compared with the reference genomes of the *Bacillus* species (Supplementary Table S1) to assess the evolutionary and phylogenetic relationships of the sequenced bacterial isolate strain NB2 with sequenced *Bacillus* genomes. The phylogenomic relationship of the bacterial isolate strain NB2 was assessed with other *Bacillus* genomes using M1CR0B1AL1Z3R webserver (https://microbializer.tau.ac.il/). The average nucleotide identity and tetra-correlation values were calculated using J-species software (http://jspecies.ribohost.com/jspeciesws/). The assembled genome was annotated with PROKKA-v1.12 annotation pipeline (Seemann, 2014). The pathogenic islands within the sequenced genome were detected with the Island Viewer 4 (https://www.pathogenomics.sfu.ca/islandviewer/resources/) following default parameters (https://www.pathogenomics.sfu.ca/islandviewer/about/). The antibiotic resistance genes were identified using the comprehensive antibiotic resistance database (CARD) (https://card.mcmaster.ca/) and ResFinder-4.1 server (https://cge.food.dtu.dk/services/ResFinder/). The dbCANmeta server (https://bcb.unl.edu/dbCAN2/blast.php) was used to identify CAZymesin, the sequenced genome.

### Biosafety Assessments of the Bacterial Isolate Strain NB2

Hemolytic activity of the bacterial isolate strain NB2 bacterial culture was assessed using the blood agar plate (5% v/v) (Barik et al., 2021). Cellular toxicity of bacterial isolate strain NB2 was assessed against Caco-2 cell lines (Dowdell et al., 2020).

### Stress Resistance Physiology

Growth of the bacterial isolate strain NB2 was assessed in gastric (pH 2.0; pepsin for 2 h) and intestinal (pH 8.0; trypsin for 6 h) conditions (AlKalbani et al., 2019). NB2 growth was also observed in the presence of the bile salts (Nami et al., 2019). The growth pattern of human gut isolate NB2 was observed in presence of salts (NaCl and KCl) and metal/metalloids [silver nitrate (0.1–2 mM), cadmium chloride (0.1–2 mM), lead acetate (0.1–2 mM), potassium dichromate (0.1–2 mM), and sodium arsenate (0–50 mM); Supplementary Table S2]. The growth pattern of the bacterial isolate strain NB2 was continuously assessed with an interval of 2 h after growing active microbial culture [0.05 OD (600 nm)] for 24 h in LB broth supplemented with a respective stressor. Resistance of the bacterial isolate strain NB2 was observed against lysozyme activity (Samedi and Linton Charles, 2019). Antibiotic susceptibility of the bacterial isolate strain NB2 was observed against antibiotic discs of amikacin, Amoxicillin, Bacitracin, Cephalothin, Erythromycin, Novobiocin, Oxytetracycline, Vancomycin, Ceflnaxone, Ceftazidime, Cefotaxime, Lincomycin, Netilin, and Ofloxacin (Himedia, OD034R-1PK, and OD003R-1PK) using disc diffusion assay after recording the zone of the growth inhibition (mm) on the LB agar medium after incubation for 24 h at 37°C.

### Auto-Aggregation and Cell Surface Hydrophobicity

The auto-aggregation tendency and cell surface hydrophobicity of the bacterial isolate strain NB2 were also observed (Collado et al., 2008; Dowarah et al., 2018).

### Health-Promoting Features of Bacterial Isolate Strain NB2

The anti-pathogenic property of the bacterial isolate strain NB2 was screened against the pathogens *Staphylococcus aureus* (MTCC No. 96)*, E. coli* (MTCC No. 443), and *Salmonella typhi* (MTCC No. 98) with a disc diffusion assay (Kumar et al., 2021). Co-aggregation tendency of the bacterial isolate strain NB2 with pathogens [*Staphylococcus aureus* (MTCC No. 96)*, E. coli* (MTCC No. 443), and *Salmonella typhi* (MTCC No. 98)] was also assessed (Valeriano et al., 2014). Human gut bacterial isolate strain NB2 was assessed for anti-amylase activity (Sekhon-Loodu and Rupasinghe, 2019), anti-lipase activity (Jaradat et al., 2020), cholesterol removal activity (Shobharani and Halami, 2016), and bile salt hydrolysis activity (Shobharani and Halami, 2016). Bacterial isolate strain NB2 was screened for the activity of glutenase (Shobharani and Halami, 2016), prolylendopeptidase (Kumar et al., 2018), lactase (Leksmono et al., 2018), laccase (Mandic et al., 2019), peroxidase (https://www.sigmaaldrich.com/IN/en/technical-documents/protocol/protein-biology/enzyme-activity-assays/enzymatic-assay-of-peroxidase), and phosphatase (Ndubuisil et al., 2002).

## Results

### Characterization of Bacterial Isolate Strain NB2

**Figure 1:**
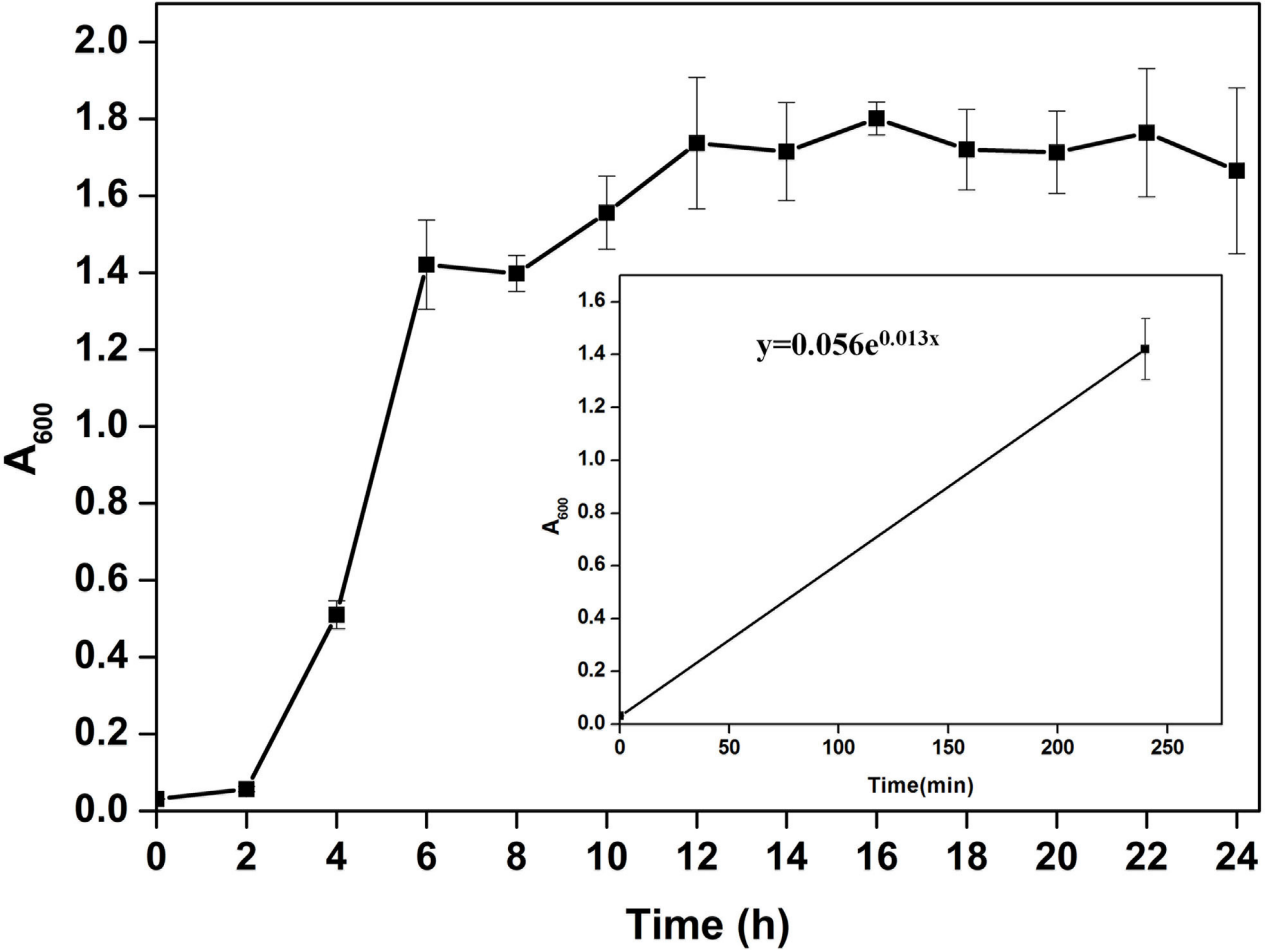
Growth pattern analysis of the bacterial isolate strain NB2 in Luria-Bertani broth for 24 h at 37°C with constant shaking at 200 rpm. Each point in the graph is the mean value of readings observed in triplicate experiments.

**Figure 2:**
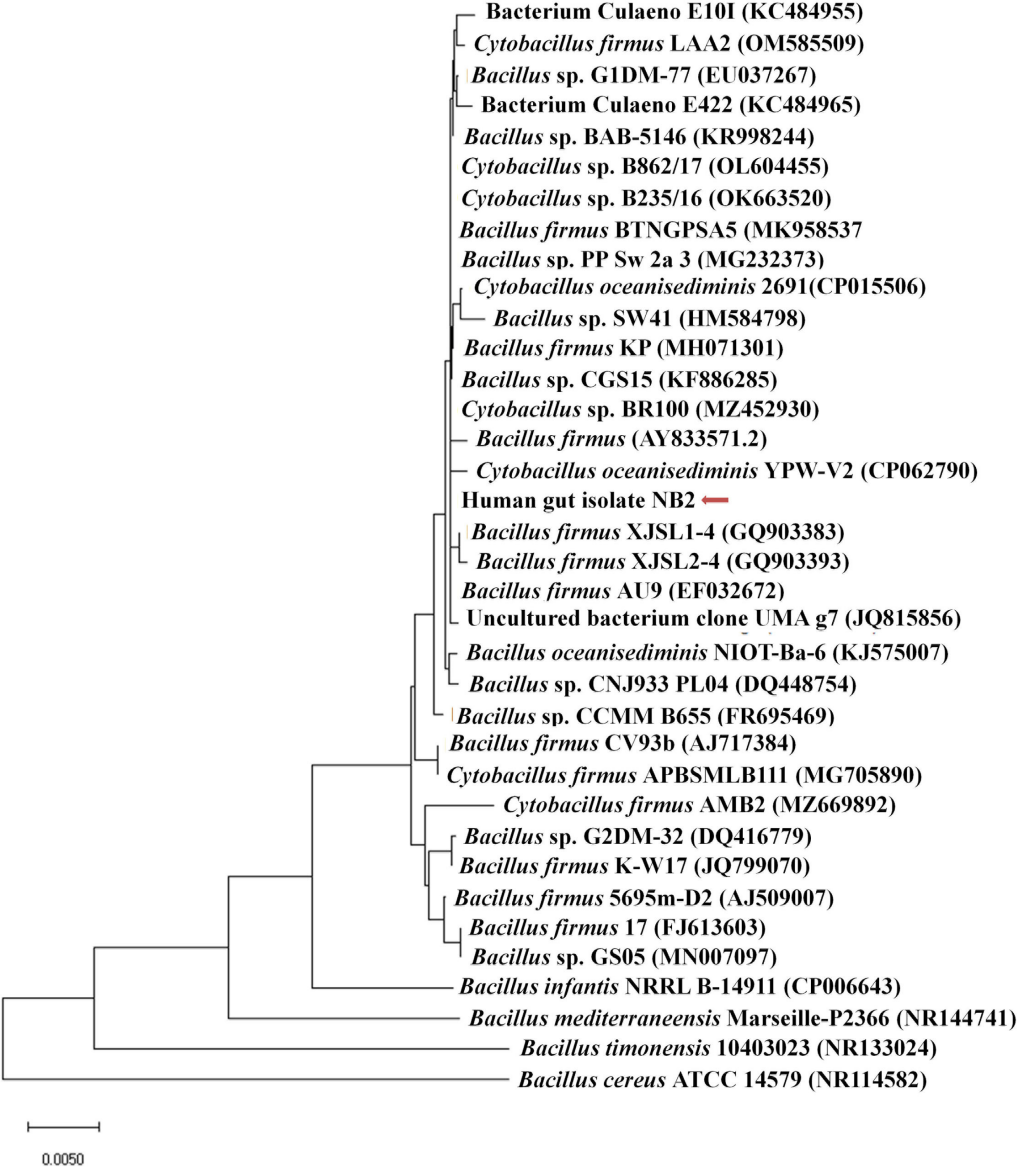
Phylogenetic affiliation of bacterial isolate strain NB2 with the other *Bacillus* species. Phylogenetic tree was constructed with the neighbor-joining method of phylogenetics using the 16S rRNA gene sequences of bacterial isolate strain NB2 and NCBI database homologs using MEGAX software. Numbers at the node represent bootstrap values in percent for the node (based on 500 bootstrap sampling). Out-group was represented by *Bacillus cereus* ATCC 14579 SSU rRNA gene sequence.

Microscopic observation of human gut isolate NB2 indicated it is a rod-shaped gram-negative bacteria. Growth pattern analysis of gut isolate NB2 indicated that this microbe attains log phase after 2 h (Figure 1), and a doubling time for the isolated gut microbe was observed to be 53.3 min in aerobic growth conditions. Additionally, bacterial isolate strain NB2 also showed growth (0.413 OD at 600 nm) after incubating the culture for 24 h at 37°C in anaerobic growth conditions. Good growth in aerobic conditions in comparison to anaerobic conditions indicates its growth preference in aerobic conditions. It also indicates its facultative nature. The 16S rRNA gene of the gut isolate NB2 shared 99 and 96% nucleotide similarity with *Cytobacillus oceanisediminis* 2691 (CP015506.1) and *Bacillus firmus* (AY833571.2), respectively (Supplementary Table S3) indicating its taxonomic affiliation with *Cytobacillus oceanisediminis*. Phylogenetic analysis of gut isolate 16S rRNA gene also indicated a similar observation (Figure 2). Combining the 16S rRNA gene homology and polygenetic analysis, bacterial isolate strain NB2 was labeled as *Cytobacillus* sp. NB2 till further taxonomic characterization. Substrate utilization assay of the human gut isolate NB2 indicated its potential to utilize xylose, maltose, raffinose, trehalose, melibiose, sucrose, L-arabinose, mannose, esculin, and citrate out of the given 35 substrates (Table 1; Supplementary Table S4). Substrate-utilization preference of gut isolate NB2 was found similar to the *Cytobacillus oceanisediminis* as compared to *Bacillus firmus* (Supplementary Table S4). Even the antibiotic susceptibility assay of gut isolate NB2 (Supplementary Table S5) indicates its higher similarity with *Cytobacillus oceanisediminis* than *Bacillus firmus*. Substrate utilization assay, antibiotic susceptibility along with 16S rRNA gene homology, and phylogenetic analysis indicate that the gut isolate NB2 is a strain of *Cytobacillus oceanisediminis*, hence labeled as *Cytobacillus oceanisediminis* NB2.

**Table 1:** Physiological, morphological, and biochemical characterization of *Cytobacillus oceanisediminis* NB2.

**Property**	**Term**
Gram stain	Negative
Cell shape	Rod
Temperature range	20–40°C
Optimum temperature	37°C
pH range	5–9
Optimum pH	8.0
Habitat	Human gut
Salinity/metal/metalloid resistance	Upto 7% NaCl and 10% KCl; Silver nitrate (upto 2 mM); Lead acetate (upto 2 mM); Sodium arsenate (upto 10 mM); Potassium dichromate (upto 2 mM)
Substrate utilization preference	Xylose, Maltose, Raffinose, Trehalose, Melibiose, Sucrose, L-arabinose, Mannose, Esculin, and Citrate
Oxygen requirement	Aerobic/Anaerobic
Biotic relationship	Host-associated
Pathogenicity	Non-pathogenic

### Genomic Characterization of *Cytobacillus oceanisediminis* NB2

**Figure 3:**
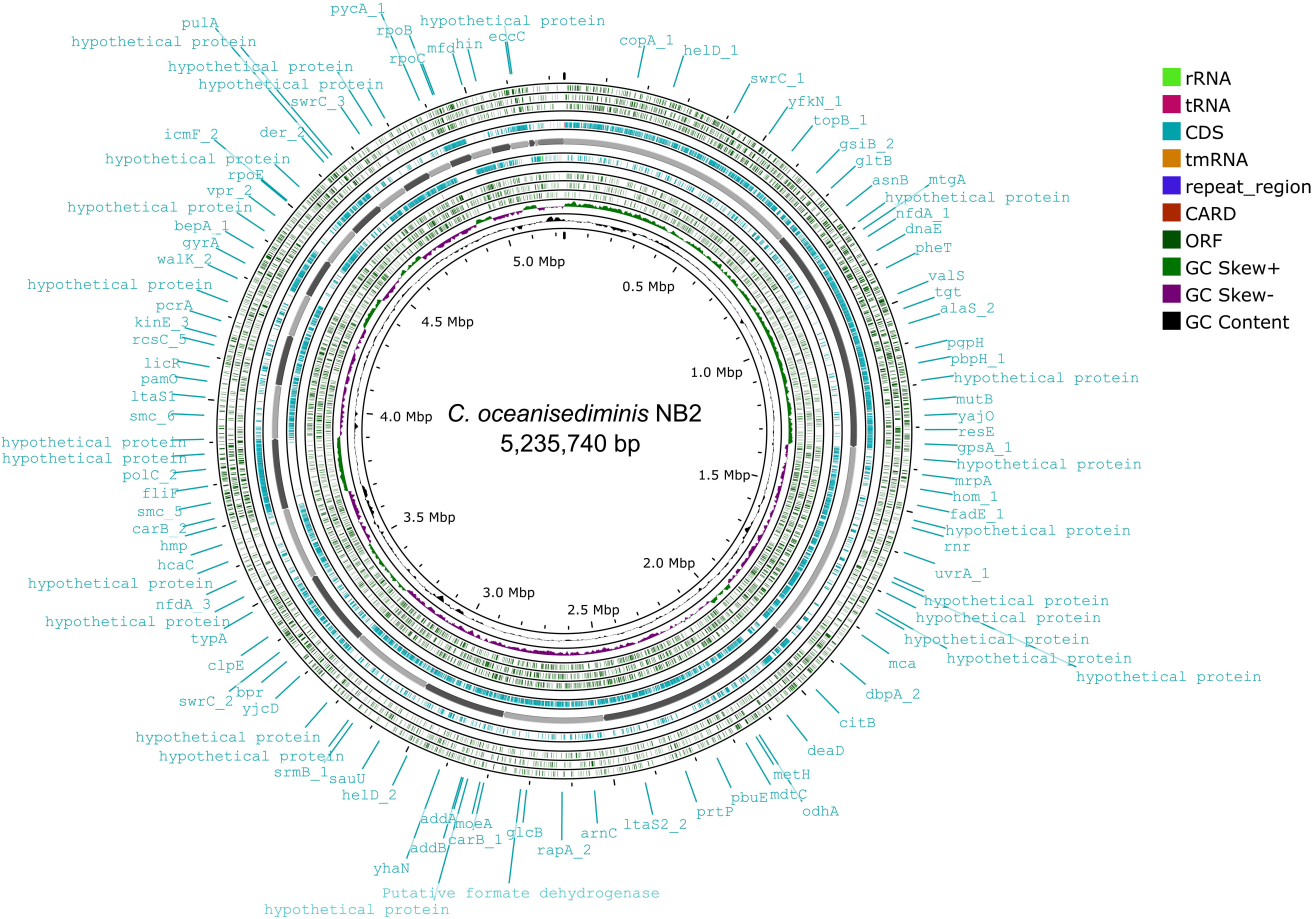
Genome map of the *Cytobacillus oceanisediminis* NB2. The circular genome map was drawn using Proksee online tool (https://proksee.ca/) that uses a complete genome sequence and annotates it using PROKKA. It also identifies resistance gene using CARD identifier.

Genome sequence assembly of *Cytobacillus oceanisediminis* NB2 resulted in 203 contigs amounting to 5,235,740 base pairs with 41.41% GC content (Supplementary Table S6). BUSCO v5.0.0 assessment tool was used with the latest bacterial ortholog catalog (bacteria_odb10) for analyzing the completeness of the set of predicted genes in the bacterial genome assembly (Supplementary Table S6). A quantitative assessment of the completeness in terms of the expected gene content of a genome assembly or annotated gene set (https://busco.ezlab.org/) was done. The BUSCO assessment resulted in 100% genome assembly and 124 complete, 123 single copies, 1 duplicated copy, 0 fragmented, and 0 missing conserved proteins within the bacterial genome. Genome annotation has identified 5,195 coding genes and 128 RNAs in the genome of *Cytobacillus oceanisediminis* NB2 (Figure 3; Supplementary Table S7). Genome characterization identified the presence of 254 genome-encoded protein features associated with metal/metalloid toxicity resistance, 147 features associated with antibiotic resistance, 414 protein features associated with oxidative stress tolerance, and 108 features associated with heat tolerance (Supplementary Table S8). Additionally, CAZymes annotation with HMMER resulted in a total of 35 CAZymes clusters (Supplementary Table S9). No pathogenic islands/genes were determined within the genome of the microbial isolate. Additionally, the virulence genes were manually searched within the genome of the microbial isolate resulting in the absence of many genes related to the pathogenic behavior of the isolated microbe.

### Genome Comparison of *Cytobacillus oceanisediminis* NB2

**Figure 4:**
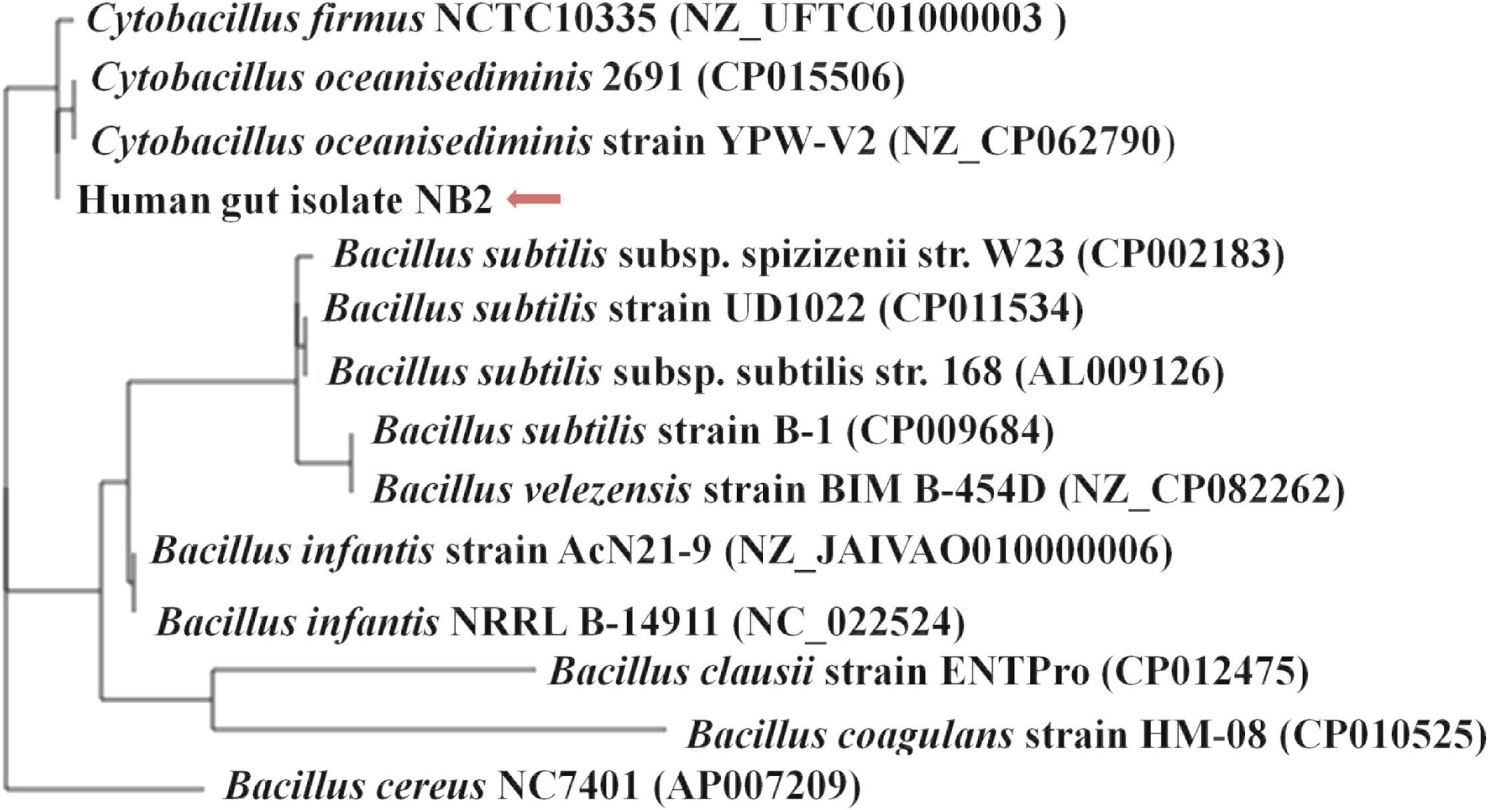
Phylogenetic assessment of the genome of *Cytobacillus oceanisediminis* NB2 with the genomes of other *Bacillus* species. The phylogenetic relationship of the gut bacterial isolate strain NB2 was assessed with other *Bacillus* genomes using M1CR0B1AL1Z3R webserver (https://microbializer.tau.ac.il/). The online tool extracts ORFs, detects OGs, extracts OG sequences, infers a core proteome, and reconstructs the species' phylogeny. The tree was drawn by taking Maximal e-value cutoff: 0.01; identity minimal percent cutoff: 80.0%; minimal percentage for core: 100.0% with no bootsrapping.

The sequenced genome of *Cytobacillus oceanisediminis* NB2 was compared with the genomes of the *Bacillus* species isolated from the various sources (Supplementary Table S1) to elucidate genome-level similarities and uniqueness. The comparison was made for genome size, coding sequences, tRNA, and rRNA (Supplementary Table S1). Additionally, the average ANI value among all *Bacillus* species was ~66–97%, which is toward the lower end of the 62–100% spectrum of interspecies variation within a genus (Kim et al., 2014), suggesting substantial genomic diversity. This observation was reaffirmed by tetra correlation among member species, highlighted by a wide distribution of *z*-scores (Supplementary Table S10). The isolated gut microbe shared high ANI (>98.0%) with *Bacillus oceansedimins* while ANIs with *Bacillus mediterraneensis, Bacillus subtilis, Bacillus clausii, Bacillus* sp. *bd59s*, and *Bacillus megaterium* were found to be 69.5867.16, 66.00, 67.25, and 68.05%, respectively (Table 2). A z-score value of 0.99806 during tetra-correlation scoring corroborates its similarity with *Bacillus oceansedimins*. Other *Bacillus* strains also shared good similarities with the gut isolate (*z*-score ~0.90–0.99; Supplementary Table S9). Genome-based phylogenetic analysis of *Cytobacillus oceanisediminis* NB2 also indicated its similarity with *Cytobacillus oceanisediminis* (Figure 4). Genome characterization indicated the presence of a total of 19736 COGs (Supplementary Figure S1). These results indicate genome plasticity, which could be attributed due to niche-specific genome evolution (Woodcock et al., 2017).

**Table 2:** Average nucleotide identity (ANI) of *Cytobacillus oceanisediminis* NB2 with reference to other *Bacillus* species.

	***Cytobacillus oceanisediminis*** **NB2 (current study)**	***Bacillus clausii*** **strain ENTPr**	***Bacillus coagulans*** **strain HM-08**	***Bacillus infantis*** **NRRLB-14911**	***Bacillus subtilis*** **strain B-1**	***Bacillus subtilis*** **strain UD1022**	***Bacillus subtilis*** **subsp. Spizizenii str. W23**	***Bacillus subtilis*** **subsp. subtilis str.168**	***Cytobacillus firmus***	***Bacillus velezensis*** **strain BIMB-454D**	***Cytobacillus oceanisediminis*** **2691**	***Cytobacillus oceanisediminis*** **strain YPW-V2**
*Cytobacillus oceanisediminis* NB2 (current study)	^*^	66.12 [17.61]	67.75 [20.05]	70.98 [39.56]	67.52 [21.36]	67.83 [23.30]	67.96 [23.27]	67.83 [23.37]	88.39 [63.37]	67.58 [21.54]	98.00 [89.18]	97.89 [90.28]
*Bacillus clausii* strain ENTPr	66.00 [20.90]	^*^	66.01 [17.64]	65.68 [19.23]	66.12 [19.35]	65.89 [21.37]	66.19 [21.03]	65.97 [21.36]	65.91 [19.63]	66.06 [19.91]	65.91 [20.96]	65.98 [21.17]
*Bacillus coagulans* strain HM-08	67.85 [28.25]	66.36 [20.27]	^*^	68.08 [28.83]	67.71 [26.92]	67.34 [27.28]	67.35 [26.65]	67.33 [27.29]	68.25 [27.98]	67.78 [27.16]	67.87 [28.37]	67.77 [28.83]
*Bacillus infantis* NRRLB-14911	71.15 [41.79]	65.81 [17.42]	68.06 [21.95]	^*^	67.78 [23.34]	67.74 [24.21]	67.74 [24.24]	67.72 [24.17]	71.41 [39.28]	67.78 [23.57]	71.18 [41.90]	71.16 [42.21]
*Bacillus subtilis* strain B-1	67.48 [27.18]	65.95 [21.06]	67.33 [24.47]	67.63 [27.57]	^*^	76.21 [72.75]	76.30 [72.81]	76.21 [73.32]	67.74 [26.31]	97.34 [93.01]	67.61 [27.32]	67.54 [27.45]
*Bacillus subtilis* strain UD1022	68.05 [29.49]	66.33 [23.29]	67.50 [25.34]	68.13 [28.66]	76.28 [72.23]	^*^	92.47 [89.08]	98.12 [94.29]	68.27 [28.03]	76.43 [72.95]	68.11 [29.75]	68.06 [29.95]
*Bacillus subtilis* subsp. *spizizenii* str. W23	68.07 [29.24]	66.24 [23.32]	67.41 [24.65]	67.71 [29.34]	76.43 [71.64]	92.60 [88.82]	^*^	92.50 [88.18]	68.17 [27.79]	76.55 [72.26]	68.06 [29.43]	68.04 [29.63]
*Bacillus subtilis* subsp. *subtilis* str.168	68.06 [28.04]	66.27 [22.16]	67.49 [24.26]	67.98 [27.50]	76.34 [69.33]	97.93 [90.56]	92.23 [85.12]	^*^	68.31 [26.49]	76.47 [71.31]	68.07 [28.40]	68.10 [28.48]
*Cytobacillus firmus*	88.56 [69.24]	66.41 [19.07]	68.52 [23.11]	71.31 [41.19]	68.11 [23.49]	68.29 [24.69]	68.27 [24.67]	68.21 [24.90]	^*^	68.07 [23.69]	88.77 [70.40]	88.71 [71.22]
*Bacillus velezensis* strain BIMB-454D	67.94 [25.78]	66.27 [20.79]	67.90 [23.63]	67.94 [26.89]	97.31 [87.52]	76.34 [69.92]	76.44 [69.43]	76.33 [71.12]	68.05 [25.22]	^*^	67.98 [26.02]	67.88 [26.24]
*Cytobacillus oceanisediminis* 2691	97.97 [85.73]	66.18 [17.40]	67.91 [19.84]	71.12 [38.50]	67.94 [20.89]	68.19 [22.72]	68.18 [22.59]	68.18 [22.74]	88.60 [61.99]	67.95 [21.02]	^*^	98.72 [87.37]
*Cytobacillus oceanisediminis* strain YPW-V2	98.02 [88.62]	66.18 [18.63]	67.87 [20.89]	71.19 [40.03]	67.82 [21.83]	68.03 [23.48]	68.11 [23.54]	68.04 [23.55]	88.65 [64.30]	67.82 [22.09]	98.81 [89.27]	^*^

* *Indicates the ANI between the same genomes*.

### Hemolytic and Cytotoxicity Activity

Evaluation of bacterial toxicity is essential before considering a bacterial strain a probiotic, as it should not be harmful to the host cells. Toxicity and hemolytic activity assessment of *Cytobacillus oceanisediminis* NB2 indicated no hemolytic activity in Blood agar plate assay after 24 h of incubation at 37°C. Even cytotoxicity analysis of *Cytobacillus oceanisediminis* NB2 demonstrated that Caco-2 cells showed 91.82 ± 5.04% and 89.28 ± 7.85% viability after 24 h of exposure with the cell-free supernatant and cell lysate of *Cytobacillus oceanisediminis* NB2, respectively.

### Stress-Response Physiology

**Figure 5:**
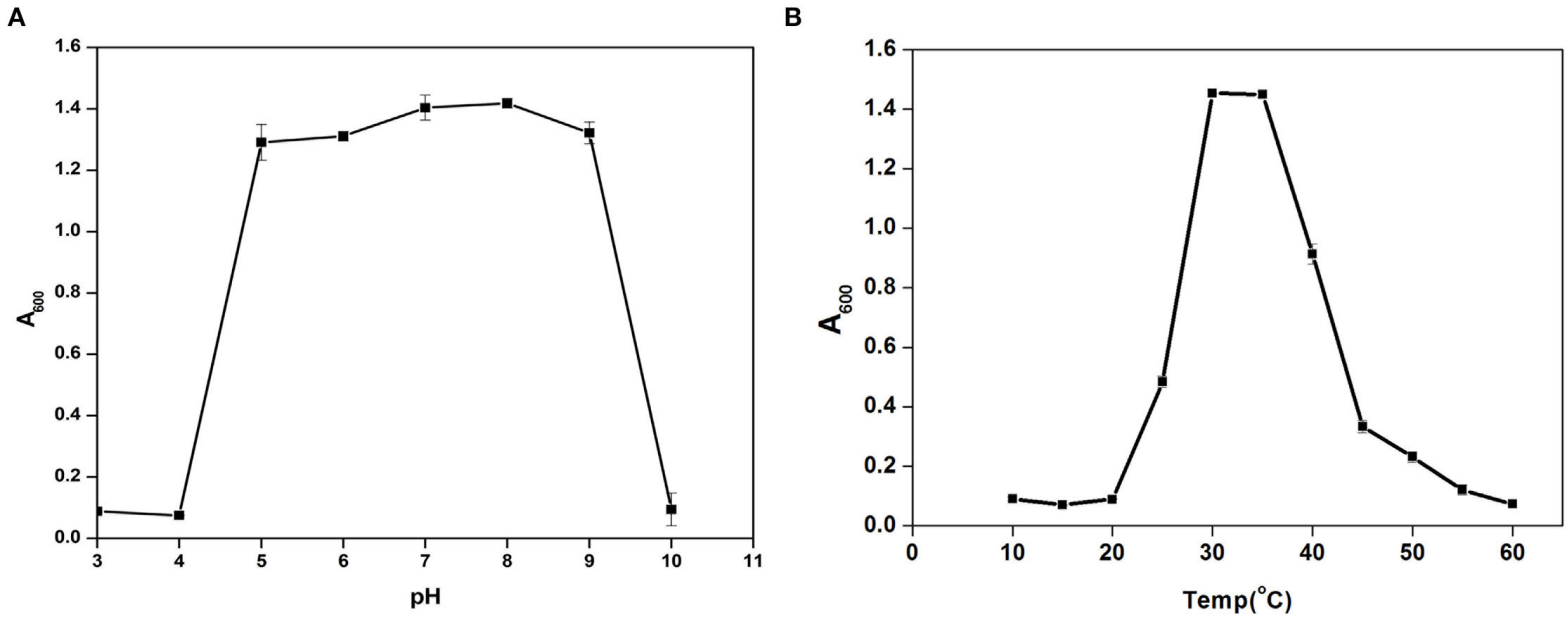
Growth pattern assessment of the *Cytobacillus oceanisediminis* NB2in Luria-Bertani broth with diverse pH (3–10 with an interval of 1.0 pH) **(A)** and temperature (10–60°C with an interval of 5°) **(B)** conditions for 24 h with constant shaking at 200 rpm. Each point in the graph is the mean value of readings observed in triplicate experiments.

*Cytobacillus oceanisediminis* NB2 showed continued growth within the pH range of 5.0–9.0, with an optimum growth at pH 8.0 (Figure 5A). *Cytobacillus oceanisediminis* NB2 showed continued growth within the temperature range of 25–50°C, while an optimum growth was observed at 30–35°C (Figure 5B). *Cytobacillus oceanisediminis* NB2 also indicated continued growth in the LB medium supplemented up to 7.0% NaCl (w/v) and 10.0% KCl (w/v). Similarly, *Cytobacillus oceanisediminis* NB2 showed growth in the presence of various metals (silver, lead, cadmium, and potassium) and metalloid arsenic. *Cytobacillus oceanisediminis* NB2 also exhibited resistance to Cephalothin (30 μg), Ceflnaxone (30 μg), Ceftazidime (30 μg), and Ofloxacin (2 μg), moderate susceptibility to Amoxicillin (10 μg), Bacitracin (10 μg), and Lincomycin (2 μg), and high susceptibility to Amikacin (10 and 30 μg), Erythromycin (15 μg), Novobiocin (30 μg), Oxytetracycline (30 μg), Vancomycin (30 μg), Cefotaxime (30 μg), and Netilin (30 μg). *Cytobacillus oceanisediminis* NB2 did not show any bile salt hydrolysis activity. Even, a 62.90 ± 0.5% growth suppression of *Cytobacillus oceanisediminis* NB2 was observed in the bile-enriched medium. *Cytobacillus oceanisediminis* NB2 did not show any growth suppression in gastric conditions, while a 48.6% growth suppression was observed in intestinal conditions. A high concentration of lysozyme (100 mg/L) suppressed the *Cytobacillus oceanisediminis* NB2 growth, while no effect was observed at lower lysozyme concentration (1 mg/L).

### Auto-Aggregation and Cell Surface Hydrophobicity

**Figure 6:**
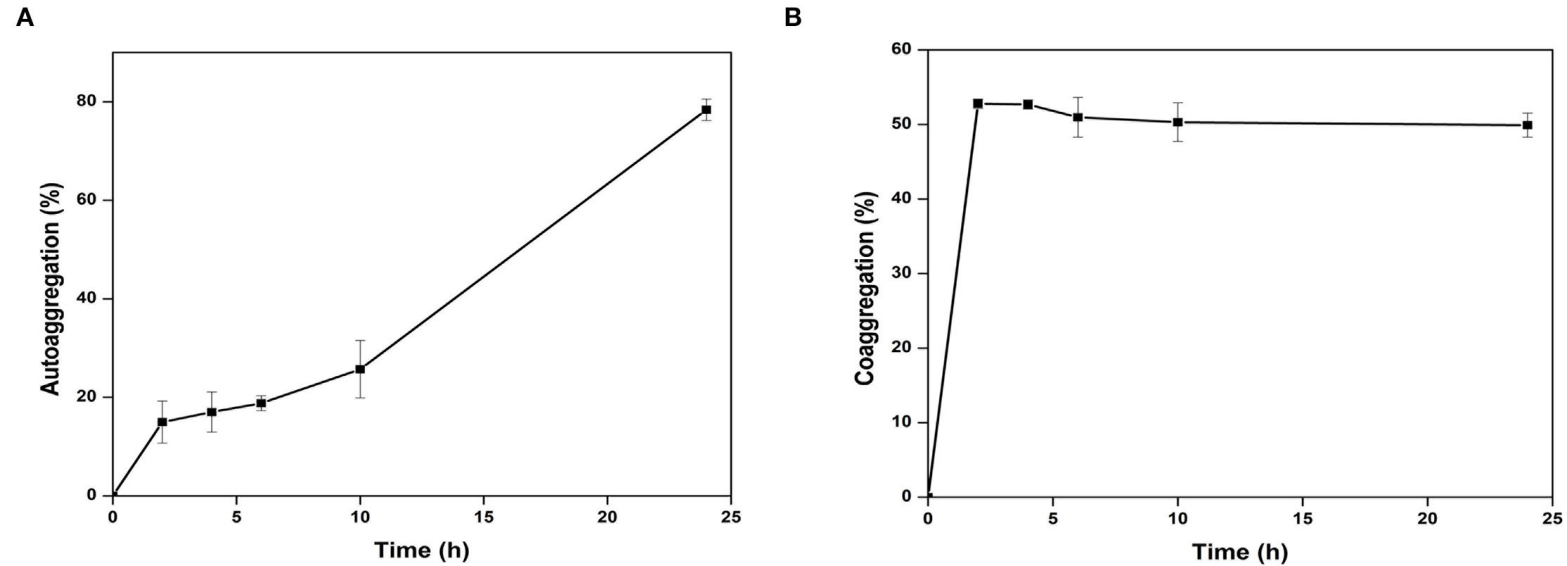
Autoaggregation **(A)** and Co-AGGREGATION **(B)** of *Cytobacillus oceanisediminis* NB2 with pathogen mixture of *E. coli, Staphylococcus aureus*, and *Salmonella typhimurium*.

The adherence properties of microbial cells are due to their aggregation abilities. The gut isolates showed adherence to the epithelial cells and mucosa due to their auto-aggregation activity (Krausova et al., 2019). *Cytobacillus oceanisediminis* NB2 cells showed low adherence to toluene (0.97% ± 0.87), which confirms the hydrophilic nature of the isolate and indicated its electron-donating nature. *Cytobacillus oceanisediminis* NB2 showed 78.43 ± 0.97% auto-aggregation after 24 h, with auto-aggregation of 15.03 ± 2.04%, 17.05 ± 2.10%, 18.82 ± 1.57%, and 25.81 ± 2.58% after 2, 4, 6, and 10 h, respectively (Figure 6A). *Cytobacillus oceanisediminis* NB2 also showed 15.89 ± 2.45 % adherence to the Caco-2 cells. Adherence to intestinal cells is an essential feature for successful establishment and colonization (Yadav and Chauhan, 2022). The cellular adherence property of *Cytobacillus oceanisediminis* NB2 indicated the possibility of its successful establishment in the gut environment.

### Health-Promoting Properties of *Cytobacillus oceanisediminis* NB2

The probiotics can modulate pathogenic abundance by co-aggregating with them (Yadav and Chauhan, 2022). *Cytobacillus oceanisediminis* NB2 was found to co-aggregate with the pathogenic strains. A time-dependent co-aggregation was observed for Staphylococcus *aureus* (MTCC No. 96)*, E. coli* (MTCC No. 443), and *Salmonella typhi* (MTCC No. 98) (Figure 6B). Disc diffusion assay showed an anti-pathogenic activity for *Cytobacillus oceanisediminis* NB2 against three pathogenic strains of *Staphylococcus aureus* (MTCC No. 96)*, E. coli* (MTCC No. 443), and *Salmonella typhi* (MTCC No. 98). A zone of 10.8 ± 1.0 mm, 12.8 ± 1.0 mm, and 13.5 ± 0.5 mm growth inhibition was observed, respectively, for Staphylococcus *aureus* (MTCC No. 96)*, E. coli* (MTCC No. 443), and *Salmonella typhi* (MTCC No. 98) that indicated *Cytobacillus oceanisediminis* NB2-induced growth inhibition.

Anti-glycemic and anti-lipogenic effects are considered therapeutic targets to overcome diabetic mellitus (Type-II), obesity, and cardiovascular pathological conditions (Salehi et al., 2020). Thus, α-amylase inhibition seems to be the prime therapeutic target. *Cytobacillus oceanisediminis* NB2 has shown 9.82 ± 0.55% inhibition in the amylase activity. The isolated microbial culture shows 14.79 ± 1.44% inhibition of the lipase activity. Similarly, the *Cytobacillus oceanisediminis* NB2 showed cholesterol-oxidizing activity. *Cytobacillus oceanisediminis* NB2 also showed a significant prolylendopeptidase activity (0.318 units/mg microbial pellet). The presence of this enzyme activity could be helpful in removing the gluten antigen to overcome gluten-induced celiac diseases. *Cytobacillus oceanisediminis* NB2 also showed a significant lactase activity (38.796 units/mg of the bacterial pellet), which could help overcome lactose indigestibility issues for lactose-intolerant individuals. *Cytobacillus oceanisediminis* NB2 was found to possess alkaline phosphatase (9.54 ± 0.04 units/mg bacterial pellet) and acid phosphatase activity (and 190.8 ± 0.16 units/mg bacterial pellet), respectively. Phosphatase activity could play an important role in cell proliferation and differentiation. This microbe was also found to have peroxidase activity (2.4804 ± 0.02 units/mg bacterial pellet) of the enzyme, which could help to overcome oxidative stress. Laccase is a multi-copper oxidase that was characterized to play a vital role in host health (Janusz et al., 2020). *Cytobacillus oceanisediminis* NB2 was also found to have laccase enzyme activity (0.004452 units/mg bacterial pellet).

## Discussion

Diet has an important impact on a healthy life (Lindefeldt et al., 2019). Supplementation of diet with probiotic strains can further augment human health (Wang et al., 2019). The application of any bacteria as a probiotic strain requires extensive characterization for safety and applicability (Yadav and Chauhan, 2022). Recently, probiotic bacteria are being extensively explored and characterized for their potential therapeutics for various human disorders (Yadav and Chauhan, 2022). Probiotic strains were identified from various sources like dairy products (Haghshenas et al., 2017; Karami et al., 2017), fermented drinks (Angelescu et al., 2019; Setta et al., 2020), plants (Rahman et al., 2018; Samedi and Charles, 2019), soil (Siraj et al., 2017), and animals (Abdou et al., 2018; Li et al., 2021). Human gut microbiota are being extensively characterized for their health-promoting benefits; however the full potential for their usage as microbiome therapeutics has humongous possibilities (Yadav and Chauhan, 2022). These characterizations are primarily performed as a consortium; however, individual-specific microbial diversity has not been characterized to date for assessing their suitability as probiotic strain, especially in different geographical regions of the world as well as within large countries like India. The lack of pure culture for a majority of gut microbes is the major bottleneck toward their functional usage (Lagier et al., 2015). Efforts are being made to culture human gut microbes in laboratory conditions to characterize them for their probiotic potential (Tang et al., 2020). Thus, the current study was planned to explore the human gut microbiota to isolate the human gut bacterium for probiotic applications.

In the current study, a bacterial culture was isolated from the human feces. Biochemical, physiological, and taxonomic characterization identifies it as a species of *Bacillus*. Members of the *Bacillus* were characterized by diverse habitats, including human feces, and showed a wide range of biotechnological potential (Singh et al., 2009; Zhang et al., 2010; Kumar et al., 2013, 2014; Patel et al., 2014; Boucherba et al., 2017). Various *Bacillus* species have already been characterized for probiotic potential (Elshaghabee et al., 2017). *B. cereus, B. clausii, B. coagulans, B. licheniformis, B. polyfermenticus, B. pumilus*, and *B. subtilis* are commercially used probiotics. Although various strains of *Bacillus* were studied in various organisms such as mice and pigs, studies regarding the *Bacillus* strains as a probiotic in the human body are still evolving (Hong et al., 2009). The bacterial isolate strain NB2 was assessed for its substrate utilization tendency where it has shown the positive esculin hydrolysis and citrate utilization that is in line with the substrate utilization characteristic of the other *Bacillus* strains (Beesley et al., 2010). The whole-genome analysis of the bacterial isolate strain NB2 indicated the presence of COGs associated with the general adaptive and metabolic mechanisms required for the microbial cell survival within the human body, thus suggesting a strong affiliation to survive and thrive within the human host (Yadav et al., 2020, 2021). The gut isolate was found to possess no pathogenic islands indicating its safety considerations for probiotic features (Li et al., 2018). The bacterial isolate strain NB2 contains a total of 35 CAZymes. The *Bacillus* strains have unique anti-cancer, anti-oxidant, anti-diabetic, as well as anti-obesity characteristics (Elshaghabee et al., 2017). Within the human body, the microbe may suffer various stressful conditions such as gastric environment, heat, temperature, and pH stress. Thus, a probiotic bacterium should possess significant features to resist all these stressful conditions. *Bacillus* strain is well-known to adapt and thrive within the host's body (Yadav et al., 2018). Likewise, *Cytobacillus oceanisediminis* NB2 was identified to thrive in high salt, variable pH, and temperature conditions indicating its suitability to survive in a highly variable gut ecosystem. Bile salts pose a major challenge to microbial survival (Bustos et al., 2018). The differential expression of bile salt resistance proteins may influence bile tolerance of the isolated microbe (Hamon et al., 2011).

*Cytobacillus oceanisediminis* NB2 showed growth in the presence of bile salt, despite slight growth suppression indicating its bile tolerance property. The strain/species-specific acid tolerance might have influenced the bacterial survival in the acidic gastric conditions (Nami et al., 2019) since certain microbial strains are adapted to thrive in acidic conditions (Guan and Liu, 2020). The *Cytobacillus oceanisediminis* NB2 did not show any growth suppression in the gastric pH in presence of pepsin while it showed partial growth suppression in the human intestinal conditions. These results indicate its survivability in diverse environments, making it suitable to apply in the human gastrointestinal ecosystem. *Bacillus* strains were known to produce toxins and may transfer antibiotic resistance; thus, a safety evaluation needs to be done (Kotowicz et al., 2019). *Cytobacillus oceanisediminis* NB2 toxicity was assessed against the Caco-2 cells. The 24-h bacterial exposure showed 89.3% cell viability, thus, indicating it is a safe and non-toxic microbe. Furthermore, the bacterial isolate strain NB2 did not show hemolysis. Auto-aggregation and co-aggregation properties are important for the anti-pathogenic potential of probiotics (Collado et al., 2008). Auto-aggregation enables the microbes to bind with each other and form the first line of defense against the pathogens (Trunk et al., 2018), while co-aggregation enables the microbial assessment for their binding capacity with the pathogens. *Cytobacillus oceanisediminis* NB2 was found to have different levels of aggregation and co-aggregation indicating differential environmental and internal factors (Vlková et al., 2008). Thus, this microbe can impart several benefits to maintain intestinal health by protecting it from pathogens. The probiotics improve the host's health without posing adverse effects on other microbial groups as well as the host due to antibiotics-led dysbiosis (Plaza-Diaz et al., 2019). The microbial factors can suppress the pathogen's survival and thus modulate the risk of infection. The gut isolate suppressed the growth of three pathogenic strains, i.e., *Salmonella typhi, E. coli*, and *Staphylococcus*. Thus, the anti-pathogenic activity indicates that microbes can be easily used to eradicate the overgrown pathogens within the host. The bacterial isolate strain NB2 was detected to possess various enzymatic activities against various substrates. Different levels of activities were obtained, and positive enzymatic activities indicate that the microbe can be used to promote the metabolic capacity of the host. The antibiotic treatment is a major threat to the host's health as it may modulate the other microbial strains as well as develop microbial dysbiosis leading to infections and health risks. The probiotics must contain the genetic features that may provide resistance to antibiotics. The bacterial isolate strain NB2 has shown resistance against a diverse range of antibiotics like (Amikacin, Amoxicillin, Bacitracin, Cephalothin, Erythromycin, Novobiocin, Oxytetracycline, Vancomycin, Ceflnaxone, Ceftazidime, Cefotaxime, Lincomycin, Netilin, and Ofloxacin). The presence of resistance against a range of antibiotics would allow it to survive in presence of antibiotic compounds. It also extends suitability when ingested with antibiotic drugs. The adherence properties of cells are due to their aggregation abilities. The bacterial isolate strain NB2 showed adherence to the epithelial cells and mucosa due to its auto-aggregation activity (Krausova et al., 2019). In the present study, the adherence ability is linked to auto-aggregation. The bacterial isolate strain NB2 cells showed low adherence to toluene (0.97 ± 0.87%) confirming the hydrophilic nature of the isolate and indicating its electron-donating nature. The bacterial isolate strain NB2 showed various health-promoting features such as anti-amylase, anti-lipase, lactase, laccase, protease, prolyl endopeptidase, and cholesterol-removing activities. Dietary polyphenols induce hyperglycemic effects by binding with the glucose transporters and inhibiting the activity of the digestive enzymes. Carbohydrate utilization by α-amylase produces glucose that causes an increase in blood glucose. Thus, α-amylase inhibition is the prime target in the case of diabetes mellitus Type-II pathophysiological condition. In the present study, *Cytobacillus oceanisediminis* NB2 has shown 9.83% inhibition in the amylase activity. Phosphatases are required for cell proliferation and differentiation (Krausova et al., 2019). The presence of alkaline, as well as acid phosphatase activities within the gut isolate indicated its significant survival within the human body. The bacterial isolate strain NB2 also possessed significant peroxidase activity that enables it to survive under oxidative stress. Lactase is required to convert lactose to glucose. Thus, the lactase enzyme is required for the treatment of lactose intolerance. The significant lactase activity within the *Cytobacillus oceanisediminis* NB2 indicated that ingestion/introduction of this microbe can be beneficial for the treatment of lactose intolerance. The presence of laccase activity indicated the role of the microbe in the digestion and metabolism of various phenolic compounds. To treat obesity, the gastrointestinal absorption of fats should be first reduced (Apovian et al., 2015). The presence of anti-lipase activity within the *Cytobacillus oceanisediminis* NB2 strongly indicates its potential for the treatment of obesity. Hypercholesterolemia is a major concern in the modern lifestyle. The removal of cholesterol from the blood can treat this disorder. The *Cytobacillus oceanisediminis* NB2 possesses significant cholesterol-removing ability. Though these initial characterizations strongly indicate the probiotic potential of *Cytobacillus oceanisediminis* NB2, further *in vivo* investigations are needed to validate its efficacy. The health-promoting tendencies of the isolated gut microbe can thus be harnessed to treat various disorders such as diabetes, lactose intolerance, hypercholesterolemia, celiac disease, as well as obesity. Microbiome engineering can thus be a significant effort for human healthcare.

## Data Availability Statement

The datasets presented in this study can be found in online repositories. The names of the repository/repositories and accession number(s) can be found at: https://www.ncbi.nlm.nih.gov/genbank/, SUB11205683.

## Ethics Statement

The studies involving human participants were reviewed and approved by Institutional Human Ethical Committee, Maharshi Dayanand University, Rohtak. The patients/participants provided their written informed consent to participate in this study.

## Author Contributions

NC designed the study and experiments. NC, MY, and RP wrote the manuscript. MY and TK carried out the experiments. MY, TK, AK, and RM did the characterization. NC, MY, AK, and RM analyzed the data. All authors edited the manuscript and approved the final draft of the manuscript.

## Conflict of Interest

The authors declare that the research was conducted in the absence of any commercial or financial relationships that could be construed as a potential conflict of interest.

## Publisher's Note

All claims expressed in this article are solely those of the authors and do not necessarily represent those of their affiliated organizations, or those of the publisher, the editors and the reviewers. Any product that may be evaluated in this article, or claim that may be made by its manufacturer, is not guaranteed or endorsed by the publisher.
