# Supplementary material for: Isolation and Characterization of Human Intestinal Bacteria Cytobacillus oceanisediminis NB2 for Probiotic Potential
Source: Front Microbiol. 2022 Jul 13;13:932795. doi: 10.3389/fmicb.2022.932795 (PMC9326467; doi:10.3389/fmicb.2022.932795)
Supplement: Supplementary file 1 [file Data_Sheet_1.PDF]

Isolation and Characterization of Human Intestinal Bacteria *Cytobacillus oceanisediminis* NB2 for probiotic potential

Monika Yadav[1], Tarun Kumar[1], Akshay Kanakan[2], Ranjeet Maurya[2,3], Rajesh Pandey[2†], and Nar Singh Chauhan[1*]

[1]Department of Biochemistry, Maharshi Dayanand University, Rohtak, Haryana, India

[2]Integrative GENomics of Host-PathogEn (INGEN-HOPE) laboratory, CSIR-Institute of Genomics and Integrative Biology (CSIR-IGIB), Mall Road, Delhi-110007, India

[3]Academy of Scientific and Innovative Research (AcSIR), Ghaziabad-201002, India

*Corresponding author

Nar Singh Chauhan (nschauhan@mdurohtak.ac.in)

Running Title: Probiotic potential of *Cytobacillus oceanisediminis* NB2

Number of Words: 7983

Number of Figures: 6

Number of Tables: 2

Supplementary Figure S1. COG categories observed within the genome of Cytobacillus oceanisediminis NB2 where R codes for General function prediction only, E codes for Amino acid transport and metabolism, C codes for Energy production and conversion, G codes for Carbohydrate transport and metabolism, J codes for Translation, ribosomal structure and biogenesis, P codes for Inorganic ion transport and metabolism, L codes for Replication, recombination and repair, M codes for Cell wall/membrane/envelope biogenesis, H codes for Coenzyme transport and metabolism, F codes for Nucleotide transport and metabolism, K codes for Transcription, T codes for Signal transduction mechanisms, I codes for Lipid transport and metabolism, O codes for Posttranslational modification, protein turnover, chaperones, V codes for Defense mechanisms, Q codes for Secondary metabolites biosynthesis, transport and catabolism, U codes for Intracellular trafficking, secretion, and vesicular transport, D codes for Cell cycle control, cell division, chromosome partitioning, S codes for Function unknown, N codes for Cell motility, and Z codes for Cytoskeleton.

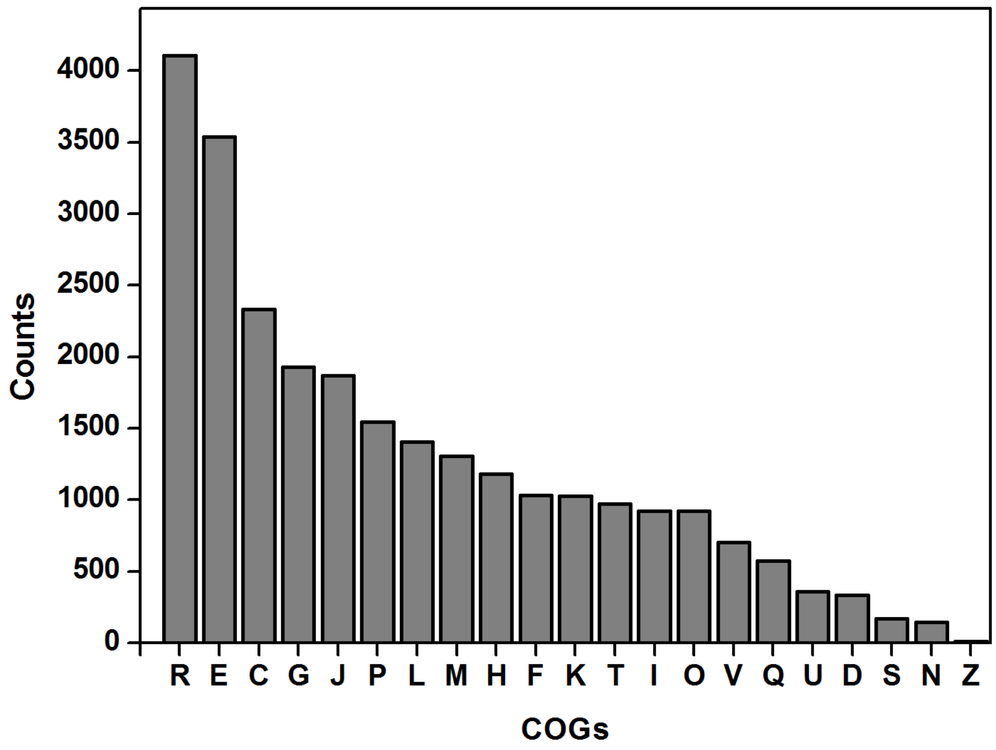

Supplementary Table S1. Genomic features of *Cytobacillus oceanisediminis* NB2 and other *Bacillus* strains used for comparative genome analysis.

Organism	Strain	Isolation source	Genome Size (Mb)	CDS	tRNA	rRNA	Genome Reference (NCBI ID)
Cytobacillus oceanisediminis	NB2	Human feces	5.2	5195	108	19	SUB11205683
Bacillus coagulans	LA204	Soil	3.37	2975	84	30	NZ_CP009709.1
Bacillus coagulans	strain HM-08	healthy Chicken intestine	3.37	2975	84	30	CP010525.1
Bacillus subtilis	strain UD1022	Root	4.02	3,933	86	30	CP011534.1
Bacillus subtilis	strain B-1	Oil field	3.94	3706	59	19	CP009684.1
Bacillus clausii	ENTPro	Soil and Water	4.44	4323	59	5	CP012475.1
Cytobacillus oceanisediminis	2691	Intertidal sediment	5.4	5679	106	33	CP015506.1
Cytobacillus oceanisediminis	strain YPW-V2	rhizosphere taken during the low tide period	5.33	5507	107	36	NZ_CP062790.1
Bacillus infantis	strain AcN21-9 AcN21-9_ctg006	sediment	5.84	4911	83	19	NZ_JAIVAO010000006.1
Bacillus firmus	NCTC10335	type strain of *Bacillus firmus*	4.8	4,436	36	108	UFTC00000000.1
Bacillus subtilis subsp. subtilis	str. 168	type strain of *Bacillus firmus*	4.22	4,237	30	86	AL009126.3
Bacillus subtilis subsp. spizizenii	str. W23	type strain of *Bacillus firmus*	4.03	3,912	24	77	CP002183.1
Bacillus velezensis	Strain BIM B-454D	Soil	4.2	4091	86	27	NZ_CP082262.1

Supplementary Table S2. A comparison of the growth conditions of *Cytobacillus oceanisediminis* NB2 with other *Bacillus* strains.

Sr. No.	NaCl	KCl	Organism	pH	Temperature (°C)	Arsenic	Cadmium chloride	Lead acetate	Silver nitrate
1	Up to 10%	ND	*Bacillus cereus* strain 01552	4.35-7.85	4.9-10	10µM	1.6308g/L	1.8307g/L	10µM
2	Up to 10%	ND	*Bacillus cereus* strain 5064	4.35-7.85	ND	ND	ND	ND	ND
3	Up to 15%	ND	*Bacillus subtilis* strain AS-4	6-8	17-37	ND	1000µg/L	1000µg/L	ND
4	Up to 10%	ND	*Bacillus cereus*	4.6-7.5	7-55	ND	ND	ND	ND
5	Up to 7%	ND	*Bacillus cereus*	4.9-9.3	4.5-55	ND	ND	ND	ND
6	0.5M	0.1M	*Bacillus subtilis*	4-9.5	Upto 60	10µM	1000µg/L	1000µg/L	10µM
7	No growth in 7%	ND	*Bacillus licheniformis*	5-9	Upto 135°C	ND	1.6308g/L	1.8307g/L	ND
8	No growth in 7%	ND	*Bacillus pumilus*	ND	ND	ND	ND	ND	ND
9	Up to 12%	ND	*Bacillus velezensis*	ND	15-45°C	ND	ND	ND	ND
10	Up to 10%	ND	*Bacillus safensis*	ND	ND	2mM	ND	ND	ND
11	ND	ND	*Bacillus subtilis* FNCC 0059	4-8.5	25-45°C	ND	1.6308g/L	1.8307g/L	ND
12	ND	ND	*Bacillus alcalophilus*	8-14	ND	ND	ND	ND	ND
13	Up to 7%	Up to 10%	*Cytobacillus oceanisediminis* NB2	5-9	20-40°C	10mM	ND	2mM	2mM

Supplementary Table S3. Nucleotide similarity of 16S rRNA gene of *Cytobacillus oceanisediminis* NB2 with other *Bacillus* strains.

Sr. No.	Matched organism	Accession Id	% identity	E value
1	*Cytobacillus oceanisediminis* 2691	CP015506.1	99	0.0
2	*Cytobacillus oceanisediminis* strain YPW-V2	CP062790.1	99	0.0
3	*Bacillus firmus*	AY833571.2	96	0.0
4	*Bacillus* sp. SW41	HM584798.1	99	0.0
5	*Bacillus firmus isolate CV93b*	AJ717384.1	99	0.0
6	*Bacillus oceanisediminis* strain NIOT-Ba-6	KJ575007.1	99	0.0
8	*Bacillus firmus* strain XJSL1-4	GQ903383.1	99	0.0

Supplementary Table S4. Carbohydrate utilization profile of *Cytobacillus oceanisediminis* NB2 in comparison to phylogenetically related *Bacillus* strains

Sr.No.	Substrate	***Cytobacillus oceanisediminis* NB2**	***Bacillus oceanisediminis* sp. nov. H2**	***Bacillus* strain YAS1**	***Bacillus clausii***	***Bacillus megaterium***	***Bacillus firmus* RAB**
1	Lactose	ND	+	+	+	+	ND
2	Xylose	+	ND	ND	+	ND	ND
3	Maltose	+	+	+	+	+	ND
4	Fructose	ND	ND	+	+	ND	+
5	Dextrose	ND	ND	+	+	+	+
6	Galactose	ND	+	+	+	ND	ND
7	Raffinose	+	ND	ND	ND	ND	ND
8	Trehalose	+	+	ND	+	ND	ND
9	Melibiose	+	ND	ND	ND	ND	ND
10	Sucrose	+	ND	ND	+	+	ND
11	L-arabinose	+	ND	ND	+	ND	ND
12	Mannose	+	+	ND	+	ND	ND
13	Inulin	ND	+	ND	+	ND	ND
14	Sodium glucan	ND	ND	ND	ND	ND	ND
15	Glycerol	ND	+	+	+	ND	ND
16	Salicin	ND	ND	ND	ND	ND	ND
17	Dulcitol	ND	ND	ND	ND	ND	ND
18	Inositol	ND	+	ND	ND	ND	ND
19	Sorbitol	ND	+	ND	+	ND	ND
20	Mannitol	ND	+	-	+	+	ND
21	Adonitol	ND	ND	ND	ND	ND	ND
22	Arabitol	ND	ND	ND	ND	ND	ND
23	Erythritol	ND	ND	ND	ND	ND	ND
24	α-Methyl-D	ND	ND	ND	ND	ND	ND
25	Rhamnose	ND	ND	ND	+	ND	ND
26	Cellobiose	ND	ND	ND	ND	ND	ND

27	Melezitose	ND	ND	ND	ND	ND	ND
28	α-Methyl-D-mannoside	ND	ND	ND	ND	ND	ND
29	Xylitol	ND	+	ND	ND	ND	ND
30	ONPG	ND	ND	ND	ND	ND	ND
31	Esculin hydrolysis	+	ND	+	ND	ND	ND
32	D-arabinose	ND	ND	ND	ND	ND	ND
33	Citrate utilization	+	ND	ND	ND	ND	ND
34	Malonate utilization	ND	ND	ND	ND	ND	ND
35	Sorbose	ND	ND	ND	ND	ND	ND

Supplementary Table S5. Antibiotic susceptibility of *Cytobacillus oceanisediminis* NB2 in comparison to phylogenetically related *Bacillus* strains.

Sr. No.	Antibiotic	***Cytobacillus oceanisediminis* NB2**	***Bacillus oceanisediminis***	***Bacillus* sp., strain YAS1**	***Bacillus clausii***	***Bacillus megaterium***	***Bacillus firmus***
1	**Amikacin**	ND	ND	ND	ND	ND	ND
2	**Amoxicillin**	ND	ND	ND	ND	ND	ND
3	**Bacitracin**	ND	ND	ND	ND	ND	ND
4	**Cephalothin**	+	ND	ND	ND	ND	+
5	**Erythromycin**	ND	ND	ND	+	ND	ND
6	**Novobiocin**	ND	ND	ND	ND	ND	ND
7	**Oxytetracycline**	ND	ND	ND	ND	+	ND
8	**Vancomycin**	ND	ND	ND	ND	+	+
9	**Ceflnaxone**	+	ND	ND	ND	ND	ND
10	**Ceftazidime**	+	ND	ND	ND	ND	ND
11	**Cefotaxime**	ND	ND	ND	ND	ND	ND
12	**Lincomycin**	ND	ND	ND	+	ND	ND
13	**Netilin**	ND	ND	ND	ND	ND	ND
14	**Ofloxacin**	+	ND	ND	ND	ND	ND

Supplementary Table S6. BUSCO assessment of genome of *Cytobacillus oceanisediminis* NB2.

Assembly	*Cytobacillus oceanisediminis* NB2 contigs
# contigs (>= 0 bp)	203
# contigs (>= 1000 bp)	30
# contigs (>= 5000 bp)	25
# contigs (>= 10000 bp)	25
# contigs (>= 25000 bp)	23
# contigs (>= 50000 bp)	23
Total length (>= 0 bp)	5235740
Total length (>= 1000 bp)	5170386
Total length (>= 5000 bp)	5163368
Total length (>= 10000 bp)	5163368
Total length (>= 25000 bp)	5135080
Total length (>= 50000 bp)	5135080
# contigs	56
Largest contig	705313
Total length	5187457
GC (%)	41.41
N50	289521
N75	204462
L50	5
L75	10
# N's per 100 kbp	0

Supplementary Table S7. Comparison of the genome features of *Cytobacillus oceanisediminis* NB2 with reference genomes of the *Bacillus* species

Genome feature	*Cytobacillus oceanisediminis* NB2	*Bacillus clausii* strain ENTPro	*Bacillus coagulans* strain HM-08	*Bacillus infantis* NRRL B-14911	*Bacillus subtilis* strain B-1	*Bacillus velezensis* strain BIM B-454D	*Cytobacillus firmus* strain NCTC10335	*Cytobacillus oceanisediminis* 2691
**Bases (Mb)**	5.23	4.44	3.37	4.88	4.22	4.01	4.80	5.63
**CDS**	5195	4446	3057	4707	4237	3761	4775	5297
**rRNA**	19	5	30	27	30	21	36	18
**tRNA**	108	59	84	86	86	81	107	77

Supplementary Table S8. Subsystems related protein features for the resistance to metal/metalloid resistance identified in the genome of *Cytobacillus oceanisediminis* NB2.

Nature of Resistance	Identified Protein Feature	No. of gene features
**Arsenic resistance**	Arsenate reductase (EC 1.20.4.1)	18
**Arsenic resistance**	Arsenic efflux pump protein	33
**Arsenic resistance**	Arsenical resistance operon repressor	4
**Arsenic resistance**	Arsenical-resistance protein ACR3	4
**Cadmium resistance**	Cadmium efflux system accessory protein	6
**Cadmium resistance**	Cadmium-transporting ATPase (EC 3.6.3.3)	65
**Resistance to chromium compounds**	Chromate transport protein ChrA	8
**Cobalt-zinc-cadmium resistance**	Cobalt-zinc-cadmium resistance protein	66
**Cobalt-zinc-cadmium resistance**	Cobalt-zinc-cadmium resistance protein CzcD	10
**Copper homeostasis:copper tolerance**	Cytoplasmic copper homeostasis protein cutC	2
**Copper homeostasis: copper tolerance**	Magnesium and cobalt efflux protein CorC	5
**Zinc resistance**	Response regulator of zinc sigma-54-dependent two-component system	2
**Cobalt-zinc-cadmium resistance**	Transcriptional regulator, MerR family	31

Supplementary Table S9. List of CAZzymes determined within *Cytobacillus oceanisediminis* NB2 genome.

Sr No.	Enzyme family	Activities in Family	Hits
1	**Carbohydrate Esterase Family 4**	Acetyl xylan esterase; Chitin deacetylase; Chitooligosaccharidedeacetylase; Peptidoglycan GlcNAcdeacetylase; Peptidoglycan N-acetylmuramic acid deacetylase	9
2	**Glycoside Hydrolase Family 43 / Subf 11**	β-1,3-xylosidase; Xylan 1,4-b-xylosidase; α-L-arabinofuranosidase	1
3	**Glycosyl Transferase Family 27**	Polypeptide α-N-acetylgalactosaminyltransferase	1
4	**Glycosyl Transferase Family 4**	Sucrose synthase; Sucrose-phosphate synthase; α-glucosyltransferase; Lipopolysaccharide N-acetylglucosaminyltransferase; Phosphatidylinositol α-mannosyltransferase; GDP-Man: Man1GlcNAc2-PP-dolichol α-1,3-mannosyltransferase; GDP-Man: Man3GlcNAc2-PP-dolichol/Man4GlcNAc2-PP-dolichol α-1,2-mannosyltransferase; Digalactosyldiacylglycerol synthase; 1,2-diacylglycerol 3-glucosyltransferase; Diglucosyldiacylglycerol synthase; Trehalosephosphorylase; NDP-Glc: α-glucose α-glucosyltransferase / α,α-trehalose synthase; GDP-Man: Man2GlcNAc2-PP-dolichol α-1,6-mannosyltransferase; UDP-GlcNAc: 2-deoxystreptamine α-N-acetylglucosaminyltransferase; UDP-GlcNAc: ribostamycin α-N-acetylglucosaminyltransferase; UDP-Gal α-galactosyltransferase; UDP-Xyl α-xylosyltransferase; UDP-GlcA α-glucuronyltransferase; UDP-Glc α-glucosyltransferase; UDP-GalNAc: GalNAc-PP-Und α-1,3-N-acetylgalactosaminyltransferase; UDP-GalNAc: N,N'-diacetylbacillosaminyl-PP-Und α-1,3-N-acetylgalactosaminyltransferase; ADP-dependent α-maltose-1-phosphate synthase; UDP-GlcNAc: polypeptide α-N-acetylglucosaminyltransferase; UDP-GlcNAc: α-N-acetylglucosaminyltransferase	11
5	**Carbohydrate-Binding Module Family 50**	Modules of approx. 50 residues found attached to various enzymes from families GH18, GH19, GH23, GH24, GH25 and GH73, i.e. enzymes	6

		cleaving either chitin or peptidoglycan	
6	**Glycoside Hydrolase Family 109**	α-N-acetylgalactosaminidase; β-N-acetylhexosaminidase	7
7	**Glycoside Hydrolase Family 13 / Subf 31**	Hexosyltransferases; Oligosaccharide a-4-glucosyltransferase; Palatinase; α-amylase; Oligo-a-1,6-glucosidase; α-glucosidase; Glucodextranase; Isomaltulose synthase /sucrose isomerase /sucrose glucosylmutase	3
8	**Glycosyl Transferase Family 28**	1,2-diacylglycerol 3-β-galactosyltransferase; 1,2-diacylglycerol 3-β-glucosyltransferase; UDP-GlcNAc: Und-PP-MurAc-pentapeptide β-N-acetylglucosaminyltransferase; Digalactosyldiacylglycerol synthase	3
9	**Glycoside Hydrolase Family 32**	Invertase; Endo-inulinase; β-2,6-fructan 6-levanbiohydrolase; Endo-levanase; exo-inulinase; Fructan β-(2,1)-fructosidase/1-exohydrolase; Fructan β-(2,6)-fructosidase/6-exohydrolase; Sucrose:sucrose 1-fructosyltransferase; Fructan:fructan 1-fructosyltransferase; Sucrose:fructan 6-fructosyltransferase; Fructan:fructan 6G-fructosyltransferase; Levanfructosyltransferase; Sucrose:sucrose 6-fructosyltransferase (6-SST); Cycloinulo-oligosaccharide fructanotransferase	1
10	**Carbohydrate Esterase Family 14**	N-acetyl-1-D-myo-inosityl-2-amino-2-deoxy-α-D-glucopyranosidedeacetylase; Diacetylchitobiosedeacetylase; Mycothiol S-conjugate amidase	3
11	**Carbohydrate-Binding Module Family 41; Glycoside Hydrolase Family 13 / Subf 41**	Modules of approx. 100 residues found in primarily in bacterial pullulanases; [retaining] α-amylase	2
12	**Glycosyl Transferase Family 51**	Murein polymerase	6
13	**Glycoside Hydrolase Family 31**	α-glucosidase; α-galactosidase; α-mannosidase; α-1,3-glucosidase; Sucrase-isomaltase; α-xylosidase; α-glucanlyase; isomaltosyltransferase; Oligosaccharide α-1,4-glucosyltransferase; α-N-acetylgalactosaminidase; Sulfoquinovosidase; α-6-glucosyltransferase	2
14	**Carbohydrate-Binding Module Family 32**	Binding to galactose and lactose	1

15	**Carbohydrate Esterase Family 9**	N-acetylglucosamine 6-phosphate deacetylase; N-acetylgalactosamine 6-phosphate deacetylase	2
16	**Auxiliary Activity Family 4**	Vanillyl-alcohol oxidase	5
17	**Auxiliary Activity Family 3**	Cellobiose dehydrogenase; Glucose 1-oxidase; aryl alcohol oxidase; Alcohol oxidase; pyranose oxidase; Glucose 1-dehydrogenase (FAD, quinone); Pyranose dehydrogenase; Oligosaccharide dehydrogenase (FAD)	1
18	**Glycoside Hydrolase Family 18**	Chitinase; Lysozyme; Endo-β-N-acetylglucosaminidase; Peptidoglycan hydrolase with endo-β-N-acetylglucosaminidase specificity; Nod factor hydrolase; Xylanase inhibitor; Concanavalin B; Narbonin	5
19	**Glycoside Hydrolase Family 13 / Subf 9**	α-1,4-glucan branching enzyme	1
20	**Glycosyl Transferase Family 5**	UDP-Glc: glycogen glucosyltransferase; ADP-Glc: starch glucosyltransferase; NDP-Glc: starch glucosyltransferase; UDP-Glc: α-1,3-glucan synthase UDP-Glc: α-1,4-glucan synthase	1
21	**Glycosyl Transferase Family 35**	Glycogen or starch phosphorylase	1
22	**Carbohydrate-Binding Module Family 68; Glycoside Hydrolase Family 13 / Subf 14**	Binding to maltotriose and maltotetraose; a-glycosidase; α-amylase; Pullulanase; Isoamylase	1
23	**Glycoside Hydrolase Family 170**	6-phospho-N-acetylmuramidase	1
24	**Carbohydrate Esterase Family 9**	N-acetylglucosamine 6-phosphate deacetylase; N-acetylgalactosamine 6-phosphate deacetylase	2
25	**Glycoside Hydrolase Family 73**	Lysozyme; Mannosyl-glycoprotein endo-β-N-acetylglucosaminidase; Peptidoglycan hydrolase with endo-β-N-acetylglucosaminidase specificity	1
26	**Glycoside Hydrolase Family 4**	Maltose-6-phosphate glucosidase; α-glucosidase; α-galactosidase; 6-phospho-β-glucosidase; α-glucuronidase; α-galacturonase; Palatinase	1
27	**Auxiliary Activity Family 6**	1,4-benzoquinone reductase	1
28	**Glycoside Hydrolase Family 171**	Peptidoglycan β-N-acetylmuramidase	1

29	**Glycoside Hydrolase Family 3**	β-glucosidase; Xylan 1,4-β-xylosidase; β-glucosylceramidase; β-N-acetylhexosaminidase; α-L-arabinofuranosidase;Glucan 1,4-β-glucosidase; Isoprimeverose-producing oligoxyloglucan hydrolase; Coniferin β-glucosidase; Exo-1,3-1,4-glucanase; β-N-acetylglucosaminidephosphorylases; β-1,2-glucosidase; β-1,3-glucosidase; Xyloglucan-specific exo-β-1,4-glucanase / exo-xyloglucanase; Stevioside-β-1,2-glucosidase; Lichenase / endo-β-1,3-1,4-glucanase; Protodioscin 26-O-β-D-glucosidase	1
30	**Glycoside Hydrolase Family 1**	β-glucosidase; β-galactosidase; β-mannosidase; β-glucuronidase; β-xylosidase; β-D-fucosidase; Phlorizin hydrolase; Exo-β-1,4-glucanase; 6-phospho-β-galactosidase; 6-phospho-β-glucosidase; Strictosidine β-glucosidase; Lactase; Amygdalin β-glucosidase; Prunasin β-glucosidase; Vicianin hydrolase; Raucaffricine β-glucosidase; Thioglucosidase; β-primeverosidase; Isoflavonoid 7-O-β-apiosyl-β-glucosidase; ABA-specific β-glucosidase; DIMBOA β-glucosidase; β-glycosidase; Hydroxyisourate hydrolase; β-rutinosidase /α-L-rhamnose-(1,6)-β-D-glucosidase; Protodioscin 26-O-Î²-D-glucosidase	1
31	**Glycoside Hydrolase Family 13 / Subf 1**	Hexosyltransferases; 4-a-glucanotransferase / amylomaltase; α-amylase; Maltotriose-producing a-amylase	1
32	**Carbohydrate-Binding Module Family 34; Glycoside Hydrolase Family 13 / Subf 20**	Modules of approx. 120 residues; Cyclic a-1,6-maltosyl-maltose hydrolase; α-glycosidase hydrolyzingpullulan, starch and g-cyclodextrin; α-amylase; Maltogenic α-amylase; Neopullulanase; Pullulanase; Cyclomaltodextrinase	1
33	**Glycoside Hydrolase Family 13 / Subf 31**	Hexosyltransferases; Oligosaccharide α-4-glucosyltransferase; Palatinase; α-amylase; Oligo-a-1,6-glucosidase; α -glucosidase; Glucodextranase; Isomaltulose synthase / sucrose isomerase / sucrose glucosylmutase	1
34	**Glycoside Hydrolase Family 13 / Subf 29**	α-glucosidase; α,α-trehalose-6-phosphate hydrolase	1
35	**Glycoside Hydrolase Family 23**	Lysozyme type G; Peptidoglycan lyase also known in the literature as peptidoglycan lytic transglycosylase; Chitinase	1

Supplementary Table S10. Tetra correlation among *Cytobacillus oceanisediminis* NB2 and other *Bacillus* highlighted by a wide distribution of z-scores

Organism name	Z-score
Cytobacillus oceanisediminis 2691	0.99806
Cytobacillus firmus NBRC 15306	0.99669
Cytobacillus firmus LK28	0.99604
Cytobacillus firmus DS1	0.99583
Cytobacillus firmus NCTC10335	0.99486
Sporosarcina globispora DSM 4	0.99381
Bacillus saganii V47-23a	0.96384
Bacillus acidicola FJAT-2406	0.95647
Mesobacillus foraminis CV53	0.95066
Bacillus sp. SG-1	0.94954
Bacillus sp. NRRL B-14911	0.94889
Bacillus infantis NRRL B-14911	0.94763
Bacillus massiliglaciei Marseille-P2600	0.94738
Bacillus glennii V44-8	0.9438
Bacillus salacetis SKP7-4	0.94353
Cytobacillus gottheilii FJAT-2394	0.94319
Fictibacillus solisalsi CGMCC 1.6854	0.94182
Peribacillus cavernae L5	0.93871
Fictibacillus enclensis NIO-1003	0.93859
Bacillus sp. FJAT-27986	0.93546
Bacillus sp. SJS	0.93284
Bacillus oleivorans JC228	0.93039
Peribacillus kribbensis DSM 17871	0.92906
Bacillus badius DSM 5610	0.92874
Peribacillus simplex P558	0.92856
Bacillus capparidis DSM 103394	0.92848
Peribacillus simplex SH-B26	0.92846
Bacillus gobiensis FJAT-4402	0.92785
Bacillus badius DSM 30822	0.92777
Bacillus badius SGD-V-25	0.92774
Bacillus badius SGD-V-25	0.92774

Bacillus badius MTCC 1458	0.92756
Bacillaceae bacterium MTCC 10057	0.92742
Quasibacillus thermotolerans MTCC 8252	0.92714
Mesobacillus campisalis SA2-6	0.92703
Bacillus sp. FJAT-22058	0.92697
Halobacillus massiliensis Marseille-P3554	0.92628
Peribacillus simplex NBRC 15720 = DSM 1321 NBRC 15720	0.92616
Quasibacillus thermotolerans SGZ-8	0.92612
Bacillus sp. Soil745	0.92599
Bacillus badius NBRC 15713	0.92578
Domibacillus antri XD80	0.92567
Domibacillus epiphyticus SAB 38	0.92535
Mesobacillus zeae JJ-247	0.92533
[Brevibacterium] frigoritolerans FJAT-2396	0.9253
Bacillus sp. FJAT-21352	0.9252
Bacillus methanolicus PB1	0.92423
Lysinibacillus odysseyi 34hs-1 = NBRC 100172	0.92342
Priestia abyssalis DSM 25875	0.92321
Siminovitchia fordii DSM 16014 = CIP 108821 DSM 16014	0.92234
Falsibacillus pallidus DSM 25281	0.92197
Jeotgalibacillus campisalis SF-57	0.92121
Bacillus sp. FJAT-27225	0.92111
Cytobacillus praedii FJAT-25547	0.92024
Bacillus badius DSM 30822	0.92777
Bacillus badius SGD-V-25	0.92774
Bacillus badius MTCC 1458	0.92756
Bacillaceae bacterium MTCC 10057	0.92742
Quasibacillus thermotolerans MTCC 8252	0.92714
Mesobacillus campisalis SA2-6	0.92703
[Brevibacterium] frigoritolerans FJAT-2396	0.9253
Bacillus sp. FJAT-21352	0.9252
Bacillus methanolicus PB1	0.92423
Lysinibacillus odysseyi 34hs-1 = NBRC 100172	0.92342
Priestia abyssalis DSM 25875	0.92321
Lysinibacillus odysseyi 34hs-1 = NBRC 100172	0.92319
Siminovitchia fordii DSM 16014 = CIP 108821 DSM 16014	0.92234
Falsibacillus pallidus DSM 25281	0.92197

Jeotgalibacillus campisalis SF-57	0.92121
Bacillus sp. FJAT-27225	0.92111
Cytobacillus praedii FJAT-25547	0.92024
[Brevibacterium] frigoritolerans FJAT-2396	0.9253
Bacillus sp. FJAT-21352	0.9252
Bacillus methanolicus PB1	0.92423
Salipaludibacillus aurantiacus S9	0.91951
Bacillus methanolicus MGA3	0.91884
Cytobacillus eiseniae FJAT-2352	0.91864
Neobacillus fumarioli NBRC 102428	0.91848
Bacillus methanolicus MGA3	0.9183
Bacillus sp. FJAT-27231	0.9171
Bacillus wudalianchiensis FJAT-27215	0.91518
Bacillus massiliigabonensis Marseille-P2639	0.91464
Bacillus sp. Leaf13	0.91381
Bacillus sp. FJAT-18017	0.91373
Bacillus sp. FJAT-21945	0.91335
Peribacillus butanolivorans DSM 18926	0.91331
Bacillus sp. Soil768D1	0.91316
Jeotgalibacillus proteolyticus 22-7	0.91296
Jeotgalibacillus soli P9	0.91272
Neobacillus mesonae FJAT-13985	0.91262
Virgibacillus indicus P2-C2	0.9125
Domibacillus iocasae DSM 29979	0.91111
Oceanobacillus damuensis PT-20	0.91098
Cytobacillus solani FJAT-18043	0.91086
Bacillus sp. SA1-12	0.91066
Peribacillus psychrosaccharolyticus ATCC 23296	0.90911
Mesobacillus subterraneus DSM 13966	0.90885
Halobacillus karajensis DSM 14948	0.90842
Mesobacillus selenatarsenatis SF-1	0.90834
Bacillus dielmonensis null	0.90825
Lysinibacillus yapensis YLB-03	0.90802
Metabacillus indicus LMG 22858	0.90801
Salipaludibacillus aurantiacus S9	0.91951
Bacillus methanolicus MGA3	0.91884
Cytobacillus eiseniae FJAT-2352	0.91864
Neobacillus fumarioli NBRC 102428	0.91848
Bacillus methanolicus MGA3	0.9183
Bacillus sp. FJAT-27231	0.9171
Bacillus wudalianchiensis FJAT-27215	0.91518
Bacillus massiliigabonensis Marseille-P2639	0.91464
Bacillus sp. Leaf13	0.91381
Bacillus sp. FJAT-18017	0.91373
Bacillus sp. FJAT-21945	0.91335
Peribacillus butanolivorans DSM 18926	0.91331

Bacillus sp. Soil768D1	0.91316
Jeotgalibacillus proteolyticus 22-7	0.91296
Jeotgalibacillus soli P9	0.91272
Neobacillus mesonae FJAT-13985	0.91262
Virgibacillus indicus P2-C2	0.9125
Domibacillus iocasae DSM 29979	0.91111
Oceanobacillus damuensis PT-20	0.91098
Cytobacillus solani FJAT-18043	0.91086
Bacillus sp. SA1-12	0.91066
Peribacillus psychrosaccharolyticus ATCC 23296	0.90911
Mesobacillus subterraneus DSM 13966	0.90885
Halobacillus karajensis DSM 14948	0.90842
Mesobacillus selenatarsenatis SF-1	0.90834
Bacillus dielmonensis null	0.90825
Lysinibacillus yapensis YLB-03	0.90802
Metabacillus indicus LMG 22858	0.90801
Salipaludibacillus aurantiacus S9	0.91951
Bacillus methanolicus MGA3	0.91884
Cytobacillus eiseniae FJAT-2352	0.91864
Neobacillus fumarioli NBRC 102428	0.91848
Bacillus methanolicus MGA3	0.9183
Bacillus sp. FJAT-27231	0.9171
Bacillus wudalianchiensis FJAT-27215	0.91518
Bacillus massiliigabonensis Marseille-P2639	0.91464
Bacillus sp. Leaf13	0.91381
Bacillus sp. FJAT-18017	0.91373
Bacillus sp. FJAT-21945	0.91335
Peribacillus butanolivorans DSM 18926	0.91331
Bacillus sp. Soil768D1	0.91316
Jeotgalibacillus proteolyticus 22-7	0.91296
Jeotgalibacillus soli P9	0.91272
Neobacillus mesonae FJAT-13985	0.91262
Virgibacillus indicus P2-C2	0.9125
Domibacillus iocasae DSM 29979	0.91111
Oceanobacillus damuensis PT-20	0.91098
Cytobacillus solani FJAT-18043	0.91086
Bacillus sp. SA1-12	0.91066
Peribacillus psychrosaccharolyticus ATCC 23296	0.90911
Mesobacillus boroniphilus JCM 21738	0.90737
Metabacillus indicus DSM 16189	0.90715
Bacillus sp. 17376	0.90669
Lentibacillus halodurans CGMCC 1.3702	0.90615
Halobacillus karajensis HD-02	0.90612
Alteribacillus bidgolensis P4B	0.90589
Sediminibacillus albus CGMCC 1.6502	0.90577
Halobacillus karajensis HD-03	0.90542

Domibacillus tundrae PAMC 80007	0.90521
Terribacillus saccharophilus DSM 21619	0.90479
Alteribacillus bidgolensis DSM 25260	0.90479
